# Do People with Disabilities Have Difficulty Finding a Family Physician?

**DOI:** 10.3390/ijerph120504638

**Published:** 2015-04-28

**Authors:** Mary Ann McColl, Alice Aiken, Michael Schaub

**Affiliations:** 1 School of Rehabilitation Therapy, Queen’s University, Kingston, ON K7L-3N6, Canada; E-Mail: aa5@queensu.ca; 2 Centre for Health Services & Policy Research, Queen’s University, Kingston, ON K7L-3N6, Canada; E-Mail: schaubm@queensu.ca

**Keywords:** primary care, disability, access to health services, equity, qualitative method, barriers, wait times

## Abstract

Primary care has been ideally characterized as the medical home for all citizens, and yet recent data shows that approximately 6% do not have a family physician, and only 17.5% of family practices are open to new patients. Given acknowledged shortages of family physicians, this research asks the question: *Do people with disabilities have particular difficulty finding a family physician?* Health Care Connect (HCC) is a government-funded agency in Ontario Canada, designed to “*help Ontarians who are without a family health care provider to find one*”. Using data from HCC, supplemented by interviews with HCC staff, the study explores the average wait time for patients with disabilities to be linked with a primary care physician, and the challenges faced by agency staff in doing so. The study found that disabled registrants with the program are only slightly disadvantaged in terms of wait times to find a family physician, and success rates are ultimately comparable; however, agency staff report that there are a number of significant challenges associated with placing disabled patients.

## 1. Introduction

Primary care has been ideally characterized as the medical home for all citizens [1], and yet recent data shows that approximately 6% do not have a family physician, and only 17.5% of family practices are open to new patients. A cornerstone of a robust public health system is access to primary and preventive care for all citizens. Increased access to primary care has been shown to decrease overall health system costs, while simultaneously decreasing morbidity and mortality, and resulting in a more equitable distribution of healthcare resources [2]. Access to primary care has become a highly politicized topic worldwide [3]. In Canada, the College of Family Physicians of Canada offers of vision of primary care as a medical home for all Canadians, with an emphasis on comprehensive, coordinated, patient-centred care [1,4]. 

Research unequivocally shows that those most likely to be without primary care tend to be marginalized and disadvantaged subsets of the population [5,6]. This subset includes people with disabilities, and in fact, the more severe the disability, the less likely individuals are to receive comprehensive care [7,8,9,10]. Access to primary care includes the ability to find a family physician, to get an appointment, to enter and use the facilities of the practice, and to receive high quality care [11]. Numerous studies have found that people with disabilities are disadvantaged in all of these areas [12,13,14,15,16]. Even when people with disabilities have a family physician, they express less confidence than their non-disabled counterparts in the quality and completeness of their care [17,18,19]. People with disabilities encounter four types of barriers in their interactions with the health care system: physical barriers, attitudinal barriers, expertise barriers and system barriers [20]. 

People with severe disabilities and chronic conditions constitute a small subset of the typical primary care caseload [21]. Although only 6% of the caseload, these patients consume 33% of a primary care practice’s resources [22]. Research on how people with disabilities are perceived by family physicians shows that doctors perceive them to be more challenging [23,24]. Physicians virtually unanimously report that disabled patients take more time and are more complex [25]. These characteristics are often incompatible with the financial incentives inherent in the compensation system for physicians, whether fee-for-service or capitation. Volume-driven compensation systems create incentives for efficiency. Some physicians have been shown to select patients based on a cost-benefit analysis, systematically excluding those most likely to represent a financial burden on the practice [26,27]. Thus patients who consistently require more than the standard 10–15 min appointment represent an economic liability. Chisholm and Stewart [28] refer to the system of physician compensation as “perverse”, since it renders the neediest patients the least attractive to a prospective provider. As Kasperski [29] puts it, “*the very patients who are most likely to benefit [from primary care] are least likely to be welcomed into a practice*”.

The profession is aware of the practice of differential selection of patients, and in recent years has gone on record to state that profit maximization at the expense of patient equity is not acceptable [29,30,31]. Guidelines exist in many jurisdictions advising physicians against this practice; however, these guidelines also typically uphold the physician’s prerogative to limit his or her scope of practice, and to interview and select patients in accordance with that scope, in spite of difficulties this may cause for patients [32,33]. The College of Physicians and Surgeons of Ontario recognizes that differential selection has the potential to be interpreted as a violation of human rights; further they acknowledge that such practices undermine the public trust in the medical profession [32]. Their guidelines recommend a first-come-first-served approach to patient recruitment within the scope of practice; however, they do not specifically prohibit differential selection, and do allow exemptions on the basis of “undue hardship”. 

In February 2008, the Ontario Human Rights Commission recommended that medical practices be required to make a public statement about whether or not they are open to new patients, and if so, then patients should be accepted on a first-come-first-served basis, until the practice is deemed “full”. Explicit criteria for the definition of a “full” practice would further contribute to transparency and equity, in the interests of fairness to all Ontarians, particularly those who are at once the neediest and the most difficult to serve. 

In response to a number of policy initiatives, the number of unattached patients in Ontario dropped from 8.8% in 2006 to 6.5% in 2009 [34]. In 2009, the Health Care Connect program was created to “*help Ontarians who are without a family health care provider (family doctor or nurse practitioner) to find one*.” Health Care Connect has a mandate to prioritize “complex-vulnerable” patients, specifically people with chronic conditions and disabilities [35]. 

The College of Family Physicians of Canada [1] shows that only 17.5% of family practices are open to new patients. Given the acknowledged shortage of family physicians in all jurisdictions, this research asks the question: *Do people with disabilities have particular difficulty finding a family physician?* The goals of this study were to answer the following questions:

1. What is the average wait time for patients with disabilities to be linked with a primary care physician compared to other patients?
2. Are patients with disabilities more challenging to link to primary care practices than patients without disabilities? What are the issues facing Care Connectors when referring people with disabilities to primary care physicians?

## 2. Methods

### 2.1. Design

The study used a mixed qualitative and quantitative approach to assess the ease or difficulty of linking some of the most complex and resource-intensive patients to family physicians.

(a) We used a retrospective review of records of the Health Care Connect program, to assess the experience of Ontarians who have a disability seeking a primary care provider.
(b) The study also involved qualitative interviews with Health Care Connect staff about the processes, challenges and outcomes of their work with disabled or chronically ill patients.

### 2.2. Sample

The sample for the quantitative portion of this study included 111,209 patients registered with the Health Care Connect program between February 2009 and June 2011. This is approximately 1% of the population, with a range between 0.2 and 3.8 across health regions in the province.

At intake, patients were prioritized for linking according to a protocol that identified them as “activity-limited”. This designation was used by Health Care Connect to identify patients in most urgent need of care. Physicians were offered a one-time cash incentive ($350) to accept these patients, and an increased rate for both capitation and fee-for-service billings for the first year. The intake protocol was administered by clerical admissions staff, using a standardized set of questions. Patients were classified as “activity-limited” if they responded positively to the following question, derived from the National Population Health Survey: *“Because of a long-term physical or mental condition or a health problem, are you limited in the kind or amount of activity you can do: At home? At school or work? In other activities, such as transportation or leisure time activities?”* On average, 26.1% (range = 16.4–35.9 across regions of the province) of registrants with Health Care Connect were classified as activity-limited. It should be noted that this is a self-report identification. For the purposes of this paper, disability is defined to correspond to the Ministry’s definition of “activity-limited”.

“Care connectors” are the key personnel of the Health Care Connect program. They are registered nurses hired by each region, to liaise between prospective patients and family physicians. There are 33 Care Connectors employed across the province. We contacted Care Connectors directly and invited them to participate in the study. The sample for the study included 23 of the 33 Health Care Connectors across the province, representing 12 of 14 regions. The obstacle to participation in the remaining two regions was a highly bureaucratic process of approval that exceeded the timelines for the interview process. Given the HCC staff are a small group, it was a condition of the programmatic ethics review that identifying information regarding the interviewees not be provided.

### 2.3. Measurement and Analysis

The quantitative data used to address the first research question were provided by the Ontario Ministry of Health and Long-Term Care (MOH-LTC), collected as part of an administrative dataset by Health Care Connect. Descriptive statistics were provided to the research team in aggregate form (tables) on registration and referral patterns by region.

The qualitative portion of the study involved semi-structured telephone interviews with the Care Connectors, lasting between 10 and 30 min. Care Connectors were asked about their experiences with physicians and patients with disabilities (compared to non-disabled patients). Questions were developed in collaboration with the lead Care Connector to ensure high quality interviews. Interview questions are found below. Interviews were conducted by two research assistants. Interview contents were audiotaped, transcribed and analyzed using N-Vivo.

1. How often do you encounter patients with disabilities looking for a family doctor? What kinds of disabilities and chronic conditions do you tend to see among your patients looking for FPs?
2. What are disabled patients typically looking for in a family physician? What are their restrictions? What are their priorities?
3. What are the barriers that you encounter when trying to link patients with disabilities with family doctors? Are these barriers different from those faced by patients who do not have disabilities? (Probe for: doctor-, patient-, and system-level barriers).
4. How successful do you feel HCC has been in connecting complex-vulnerable patients with family doctors? What about patients with disabilities in particular? What aspects of the program do you think have been most successful/unsuccessful?
5. How do you feel the program could be improved to be more successful in linking disabled patients with doctors?

The study was conducted in accordance with the Declaration of Helsinki, and the protocol was approved by the Queen`s University Health Sciences Research Ethics Board, as well as by the Community Care Access Centres administering the program (Project #731743-883-2008-1012).

## 3. Results

### 3.1. Data from Health Care Connect

Over the period studied (February 2009–June 2011; 16 months), Table 1 shows that 140,380 people registered with HCC seeking a family doctor, of whom 29,171 (20.8%) were disabled. Almost two-thirds (63.8%) of the non-disabled registrants were referred to a family physician. For disabled registrants, the record is 4% better, with 67.8% referred. Looking at the four main regions of the province, there are notable differences between regions. The Central region is a large metropolitan area, including Toronto and several other major urban and suburban areas. Although participation in the program is relatively low given the very large population base, the referral rate for disabled registrants (83.2%) was almost 15% better than for non-disabled registrants (68.4%). By contrast, the North, characterized as rural and remote, had the lowest referral rates for both disabled (48.3%) and non-disabled (53.2%), and a poorer rate for disabled than non-disabled (−4.9%). The Eastern and Western regions are a mixture of moderate-sized urban and rural communities. The Western region had higher referral rates than the East, but both were about 4% more success in placing their disabled registrants. The final column shows that the overall odds of being successfully linked with a family physician are slightly better for disabled registrants in the program than for non-disabled. The only region in which this is not the case is the North. 

**Table 1:** Success rates in linking non-disabled and disabled registrants with family physicians by region.

Region ^1^	Non-Disabled			Disabled			OR
	Registered	Referred	%	Registered	Referred	%	
CENTRAL	27,232	18,625	68.4	6213	5172	83.2	1.216
EAST	35,545	22,278	62.7	9011	6239	69.2	1.103
WEST	16,443	13,059	79.4	4680	3888	83.1	1.047
NORTH	31,989	17,022	53.2	9267	4476	48.3	0.908
TOTAL	111,209	70,984	63.8	29,171	19,775	67.8	1.063

**^1^** Regions correspond with Local Health Integration Networks (LHIN) in Ontario Canada. Central region includes: Central, Toronto Central, Mississauga-Halton, Central West, Waterloo-Wellington, Hamilton-Niagara-Haldimand-Brant. Eastern region includes: Central East, Southeast, Champlain. Western region includes: Erie-St.Clair, South West. Northern region includes: North Simcoe-Muskoka, North East, North West.

Table 2 shows the wait-time to referral for disabled registrants *vs.* non-disabled, by region. The average wait time across the province was 64 days for non-disabled registrants, and only one day more for disabled—65 days. The longest wait times in the province were found in the North (103 days for non-disabled, 113 for disabled). The wait times in the rest of the province were relatively comparable, averaging between 43 and 57 days. The lowest rates were for disabled registrants in the Central region, and the highest were for disabled registrants in the Eastern region. 

**Table 2:** Wait time to link disabled *vs*. non-disabled patients with family physicians.

Region	Non-Disabled		Disabled		Difference	
	Mean (SD)	90th%-ile	Mean (SD)	90th%-ile	% ^1^	Days ^2^
CENTRAL	52 (±48.94)	142	43 (+35.5)	108	0.76	−9 *
EAST	56 (±62.77)	172	57 (+63.33)	174	1.02	+1 *
WEST	45 (±46.11)	130	49 (+48.86)	139	1.09	+4 *
NORTH	103 (+97.33)	282	113 (+108.16)	312	1.11	+10 *
MEAN	64 (+64.58)	183	65 (+70.42)	195	1.02	+1 *

**^1^** Mean (disabled)/Mean (non-disabled); **^2^** Mean (disabled) − Mean (non-disabled); * *p* < 0.05.

The % Difference column compares the average wait time for disabled and non-disabled registrants. On average, disabled registrants wait 1 day longer (or 2% longer) for a family physician than non-disabled. The greatest difference in average wait time between disabled and non-disabled is in the North, where disabled registrants wait 10 days (or 11%) longer than non-disabled to be connected to a family physician. 

The 90th percentile values show the wait times for registrants who were most challenging to link with a family physician. These rates vary from 108 to 312 days. The Central region appears to have made special efforts on behalf of disabled registrants, showing wait times at the 90th percentile considerably lower than non-disabled (108 *vs.* 142 = −36). The remaining 3 regions had longer waits at the 90th percentile for disabled registrants (East +2; West +9; North +30). Again the challenges of the Northern region seem significantly greater than any of the 3 southern regions.

### 3.2. Interviews with Health Care Connectors

#### 3.2.1. Who are Disabled Registrants?

The next section presents the qualitative results of interviews with 23 Care Connectors across the province. The results are organized to correspond with the interview questions, and illustrated where possible with quotes from respondents. The first question in the interview with Care Connectors asked them how frequently they saw disabled patients looking for a family doctor. Their responses varied from “daily” to “infrequently” or “not very often”.

The Care Connectors tended to identify disabled registrants in terms of their diagnoses. By far the most frequently mentioned condition was multiple sclerosis, followed by a number of other neurological disorders, such as, fibromyalgia, Parkinson’s disease, spinal cord injuries and brain injuries. A number of participants mentioned back injuries, degenerative joint disease and chronic pain. There was also a subset of registrants classified as disabled who had mental illness. Autism and developmental disability were mentioned by several interviewees. Some disabled registrants were also identified in terms of functional limitation—for example, the fact that they were unable to get around, used a wheelchair, or had a sensory deficit, such as hearing or visual loss. 

#### 3.2.2. What are Disabled Registrants Looking For?

Next we asked Care Connectors what they perceived disabled registrants were looking for. Some of the registrants’ stated preferences were similar to those of the non-disabled seeking primary care—they were looking for things like proximity to their home, size or location of the practice, either a male or female doctor, or a doctor of a certain age. 

Other preferences clearly had to do with the disability and special considerations that registrants felt they needed from their family physician. First and foremost, registrants with mobility disabilities sought physicians whose offices were accessible. Issues like stairs, elevators, ramps, and other obstacles were essential considerations, particularly for registrants who used a wheelchair or scooter. 

A second consideration for disabled registrants seeking a family doctor was that the doctor should have a basic understanding of the patient’s particular condition, and preferably prior experience with another patient with a similar condition. A third consideration is the willingness to fill out forms, sign or authorize applications for benefits. Many disability benefits require the authorization of a physician to certify that the patient qualifies for the benefit—for example, disabled parking permits, disability tax credit, disability insurance claims, assistive devices coverage, maintenance of driver’s license, or fitness to return to work. 

There were a number of special accommodations that disabled registrants sought from their family physicians, and they wanted to know from the outset that the doctor was amenable to these requirements. A number of registrants acknowledged that they needed more time, more attention, more coordination than their non-disabled counterparts, and wanted a doctor who was at least in principle open to these needs. Many wanted the option for home visits, telephone consults and prescription renewals. Several prospective registrants noted that they used pain medications, and wanted a physician who was prepared to treat pain aggressively. Finally, registrants indicated that they wanted a physician who was well connected with a web of specialists, and able to get them timely specialist appointments when needed.

#### 3.2.3. What are the Barriers to Finding Family Doctors for Disabled Registrants?

The third question to Care Connectors was about the barriers they faced in attempting to connect disabled registrants with a suitable family physician. The barriers could be classified as originating with the registrant, with the physician or with the system. 

The most prevalent registrant-level barrier was cost, particularly the cost of transportation to appointments. This was particularly an issue in Northern and rural regions, where distances could be great, and public transportation was not an option. Even in urban areas, registrants with limited financial resources often had no independent means of transportation, and travelling by taxi was prohibitively expensive. If accessible public transportation was available, it came with its own set of complications, such as the need to book days in advance, unpredictable delays, and geographic boundaries. Even if registrants had benefits (such as Ontario Disability Support Program, ODSP) to assist with transportation, they had to pay out-of-pocket and wait to be reimbursed. Thus for registrants with disabilities, the necessity of a doctor in close proximity to their home became more of an issue. 

Another difficulty on the registrant side related to non-disclosure of the disability upon intake. One manifestation of this is registrants who quite-rightly make a distinction between their health and their disability. They may report that they are in good health, despite the fact that they have a disability and have extraordinary primary care needs. In so doing, they lose their priority for consideration under HCC. Registrants may also not disclose the disability because they think it will disadvantage them; prospective physicians may become aware of their complicated medical history and refuse to take them, therefore they keep the disability to themselves initially. 

On the physician side, there were also a number of barriers to making timely links with prospective registrants. First and most obviously, some doctors claimed that they could not take any patients with particular types of impairments because their practices are simply not set up to accommodate them—there were physical or other types of barriers that made the practice inaccessible. Although this is potentially a human rights violation, very few patients are likely to make a human rights complaint as a way of beginning a new relationship with a doctor. Physicians’ offices have not historically been subject to public sector accessibility standards, and so can often be located in premises that are not accessible to patients with disabilities. 

The Care Connectors substantiated that some doctors explicitly state that they will not take certain kinds of patients in their practice. Most frequently these “undesirable” patients are characterized as those that take too much time to be financially viable, or those who have chronic pain or are mentally ill. Care Connectors almost unanimously identified patients needing narcotics for pain as the most difficult to link. Virtually every Connector interviewed reported the experience of doctors stating that they would only take uncomplicated patients, making it exceptionally difficult to link registrants with chronic diseases or disabilities:

“*Physicians in general want young, healthy patients, because it is easy to fit them into the 15 min appointment, and they can leave feeling good about themselves at the end of the day.”*

*“Despite* *their practice being open, doctors will either say that their workload is too heavy, or that they just won’t accept them.”*

As one Care Connector put it, as long as doctors can refuse to take specific registrants, those who need medical care the most will be the hardest to place, and Care Connectors will experience push-back on the most complicated cases.

Some physicians were explicit with Care Connectors that they did not wish to take on registrants with high administrative needs, such as those needing authorizations and forms completed. Ontario Disability Support Program (ODSP) forms in particular were mentioned by physicians as being a barrier to taking on new patients. Physicians stated that they would not take on a new patient if they were a recipient of ODSP benefits. 

A final barrier relating to physicians in some areas was low subscription rates. It is voluntary for physicians to sign up with Health Care Connect, and in some regions, Care Connectors estimated that as few as 15% of physicians were enrolled with the program. Low enrollment rates are doubly challenging, since these physicians then end up taking on very demanding registrants. A quote from one interviewee illustrates:

*“The* *doctors who are accepting patients get all the heavy cases. What [the doctors] believe about HCC is coming true. Doctors refer the patients they don’t want to take to HCC, and then we get all the patients other doctors don’t want. The doctors are making it true.”*

According to Care Connectors, there are also a number of systemic barriers to successfully linking disabled registrants with appropriate family physicians. One of the key impediments was the fact that the intake form does not adequately identify people with disabilities. The algorithm for prioritizing registrants for immediate linkage does not flag those with disabilities, so although they have many complex health needs, they may “*fall through the cracks in the system*”. Furthermore the staff administering the intake checklist are not necessarily health professionals, and so perhaps do not pick up subtle signals, probe for more information, or interpret the sub-text of patients’ needs. Care Connectors felt that registered nurses with experience in primary care would perform this function more effectively.

Another systemic barrier is the geographic distribution of doctors and of practice types. The North presents a number of geographic challenges, simply by virtue of the huge land mass and sparse population. Care Connectors were clear that disabled registrants would fare best in multi-disciplinary practice configurations, such as Family Health Teams or Community Health Centres; however, these were not always available in the vicinity of the registrant. These types of practices tend to be clustered in urban centres and in the south. Closed practices, physician retirements, specialized practices and regional shortages all conspired to make the task of linking disabled registrants more difficult.

### 3.3. Successes of HCC

When asked about the successes of the Health Care Connect program, Care Connectors were proud to report the benefits they felt they were achieving. They were proud of their record of linking registrants, particularly registrants with high needs, to the best available care in their region. They responded strongly to the advocacy role, and derived satisfaction from “*improving things for people who couldn’t advocate for themselves”.*

Care Connectors also felt they had good relationships with the physicians in their area, and were able to assist them to develop a balanced and satisfying practice. They took pride in having their fingers on the pulse of the primary care needs and resources in their communities, and working effectively with the physicians. They felt that the monetary incentives to physicians for taking patients with higher needs had helped them to do their job.

### 3.4. Improvements/Recommendations for HCC

The final question of the interview dealt with improvements that the Care Connectors might recommend. Their comments fell into three categories: physician enrollment, public awareness and intake process. 

The most universal recommendation from the program staff related to the need to enroll more doctors. Care Connectors noted that the proportion of an area’s physicians enrolled in the program differed dramatically from region to region, and even between communities within the same region. Some felt that more information and advertising was needed for the doctors to better inform them about the program. Others pointed to the success of financial incentives and suggested that these be enhanced and better publicized among physicians. Several suggested that physician enrollment should not be voluntary (as it is now), but rather that all physicians with “open” practices should be listed with the program. Along with this was a suggestion that physicians who routinely refused to take difficult patients be referred to the College to ensure accountability and human rights. 

A second improvement recommended by the Care Connectors was to increase public awareness of the program. They felt there were many more people who could be helped, and they encountered a high degree of ignorance about the program in their day-to-day conversations with members of the public.

Finally, they recommended that the intake process should be reviewed and streamlined, as they felt there were cracks where high needs patients, such as disabled patients were falling through. One respondent suggested that some of the more common disabling conditions, such as MS or cerebral palsy, should routinely be designated as a priority. Others suggested that the intake process should be staffed by health professionals who would be more aware of the terminology and needs that people might be expressing when they called in.

## 4. Discussion

The results of this analysis of data from the Health Care Connect program in Ontario Canada show that disabled registrants experience similar wait times to non-disabled when attempting to find a family physician—on average 65 days for disabled *vs*. 64 days for non-disabled registrants. In fact, disabled registrants were slightly better off in terms of success rate for linking with a family physician—67.8% *vs*. 63.8% for non-disabled registrants. Southern and central regions had higher success rates than the North for linking disabled registrants with family doctors. This finding highlights the many challenges of providing health services in the North—a huge geographic area, with a small population widely dispersed, often on aboriginal reserves (where jurisdiction for health is federal *vs*. provincial). In addition, physician human resources are scarce in the North, secondary and tertiary health services are even scarcer, and transportation can be particularly challenging, especially in winter.

The Health Care Connectors outlined with considerable consistency the particular challenges faced in trying to link disabled registrants with family physicians. They identified three types of barriers—at the patient level, the physician level and the system level. The most challenging barriers were physician issues. It should be noted that physician participation in the program was voluntary, so rates of physician participation varied significantly from community to community. Also physician human resource supply and distribution varied by region, with greater supply and concentration in urban areas, such as the Central region, and lowest in the North. 

Physicians were often reluctant to accept disabled patients, particularly those with mental illness or pain. Often these registrants had already been refused by other doctors in the region, and Health Care Connect was their last resort for finding a family doctor. Not all physicians in a regions were necessarily registered with the program, since participation in HCC is voluntary for physicians. The interviewees suspected that because HCC tended to get the more difficult patients, some physicians may have chosen not to register with the program, as a means of controlling the demands of their caseload. Even those physicians who were registered with the program often refused the registrants who were most difficult to place. 

Two limitations should be noted in interpreting the findings of this study. First, registrants with disabilities were identified by self-report in an intake interview. This approach is known to underestimate the true prevalence of disability for a number of reasons associated with reluctance to claim the label [36]. Secondly, quantitative data were received in aggregate form from the Ontario Ministry of Health and Long-term Care. Demographic data on HCC program registrants were not available. Neither were details of the Care Connector interviewees—this was a condition of the institutional ethics review. 

The literature is clear that there are inequalities in health and health care for disabled patients [8,13,19,37,38,39]. If primary care is to fulfill a meaningful role in public health, it is essential that the neediest patients are able to secure a place in a family medicine practice. Physical barriers to disabled patients, such as stairs, doorways, elevated examining tables, are the most obvious, and arguably the easiest to eliminate. Family physicians have been shown to be resourceful and adaptive in their attempts to improve access despite physical barriers Typical accommodations include seeing patients at an alternate accessible site, examining patients in their wheelchairs, rather than transferring to an examining table, making home visits, communicating by phone, email or skype [25,33]. 

Accessibility and human rights legislation in most jurisdictions is aimed at ensuring this most basic aspect of access; and yet, as Pharr [33] notes, even where practice administrators are aware of the legislation, there is no assurance that accessibility standards will be met. Osmun, Chan and Solomon [15] outline medical, legal and ethical considerations associated with providing primary care to people with disabilities. They advocate for more research on the long-term health needs of disabled individuals living in the community.

More challenging by far are attitudinal and knowledge barriers [40]. The literature is unanimous that physicians are inadequately prepared to deal with disabled patients and with disability issues in practice [16,39,41,42]. As an example, Junius-Walker and colleagues [24] found that physicians were ill-disposed toward problems associated with disability, particularly those to which there is no immediate medical solution. Many disability-related problems provoked a fatalistic attitude in the attending GP, rather than a constructive problem-solving approach [7]. 

A number of authors and advocates have responded to this gap with guidelines and educational materials [43]; however the evidence suggests that knowledge products alone are insufficient to produce the kind of change needed to overcome systemic and physician barriers. Most effective appear to be experiential learning opportunities that bring medical trainees into contact with disabled people, in an environment characterized by collaboration and appropriate supervision [41,42].

## 5. Conclusions

In conclusion, if the vision of primary care as a “medical home” is to be achieved [1,4,44], there is still work to be done in ensuring appropriate care for people with disabilities. Future studies should address the issue of severity of disability and care-seeking behavior. The Health Care Connect program appears to be succeeding “against the odds” in placing some of Ontario’s most disadvantaged individuals with family physicians in their regions. Connection rates are comparable between disabled and non-disabled registrants in the program, and Health Care Connect staff experience commendable success in developing collaborative relationships with physicians in the region, prioritizing the most difficult registrants for sustained attention, and invoking creativity and persistence in seeking to assist disabled Ontarians overcome the barriers to finding a family physician.
